# Changes of the Brain Causal Connectivity Networks in Patients With Long-Term Bilateral Hearing Loss

**DOI:** 10.3389/fnins.2021.628866

**Published:** 2021-07-01

**Authors:** Gang Zhang, Long-Chun Xu, Min-Feng Zhang, Yue Zou, Le-Min He, Yun-Fu Cheng, Dong-Sheng Zhang, Wen-Bo Zhao, Xiao-Yan Wang, Peng-Cheng Wang, Guang-Yu Zhang

**Affiliations:** ^1^Department of Otorhinolaryngology and Head-Neck Surgery, The Second Affiliated Hospital, Shandong First Medical University, Tai’an, China; ^2^Department of Radiology, The Second Affiliated Hospital, Shandong First Medical University, Tai’an, China; ^3^Department of Radiology, Shandong First Medical University & Shandong Academy of Medical Sciences, Tai’an, China

**Keywords:** auditory-visual reorganization, the shortest causal connectivity path, auditory- visual inhibition, the virtual digital brain, hearing loss, speech processing

## Abstract

It remains poorly understood how brain causal connectivity networks change following hearing loss and their effects on cognition. In the current study, we investigated this issue. Twelve patients with long-term bilateral sensorineural hearing loss [mean age, 55.7 ± 2.0; range, 39–63 years; threshold of hearing level (HL): left ear, 49.0 ± 4.1 dB HL, range, 31.25–76.25 dB HL; right ear, 55.1 ± 7.1 dB HL, range, 35–115 dB HL; the duration of hearing loss, 16.67 ± 4.5, range, 3–55 years] and 12 matched normally hearing controls (mean age, 52.3 ± 1.8; range, 42–63 years; threshold of hearing level: left ear, 17.6 ± 1.3 dB HL, range, 11.25–26.25 dB HL; right ear, 19.7 ± 1.3 dB HL, range, 8.75–26.25 dB HL) participated in this experiment. We constructed and analyzed the causal connectivity networks based on functional magnetic resonance imaging data of these participants. Two-sample *t*-tests revealed significant changes of causal connections and nodal degrees in the right secondary visual cortex, associative visual cortex, right dorsolateral prefrontal cortex, left subgenual cortex, and the left cingulate cortex, as well as the shortest causal connectivity paths from the right secondary visual cortex to Broca’s area in hearing loss patients. Neuropsychological tests indicated that hearing loss patients presented significant cognitive decline. Pearson’s correlation analysis indicated that changes of nodal degrees and the shortest causal connectivity paths were significantly related with poor cognitive performances. We also found a cross-modal reorganization between associative visual cortex and auditory cortex in patients with hearing loss. Additionally, we noted that visual and auditory signals had different effects on neural activities of Broca’s area, respectively. These results suggest that changes in brain causal connectivity network are an important neuroimaging mark of cognitive decline. Our findings provide some implications for rehabilitation of hearing loss patients.

## Introduction

Behavioral studies have reported that individuals with early or congenitally hearing loss are highly reliant on their remaining intact sensory modalities to interact with their surrounding environment (Merabet and Pascual-Leone, 2010) and displayed superior visual behavioral performing skills compared with normal-hearing controls, such as enhanced peripheral attention to moving stimuli (Neville and Lawson, 1987; Bavelier et al., 2000; Proksch and Bavelier, 2002; Dye et al., 2009) and increased performance abilities in visual motion detection (Hauthal et al., 2013; Shiell et al., 2014, 2016). Subjects with early hearing loss also exhibited faster and more accurate visual working memory performance compared with normal-hearing controls (Ding et al., 2015). However, some disadvantages of early or congenitally hearing loss on visual functions have also been reported; for example, early auditory deprivation results in visual selection deficits (Smith et al., 1998; Horn et al., 2005; Quittner et al., 2010) but does not contribute to better or worse visual attention. Selected aspects of visual attention of early hearing loss individuals are able to be modified in various ways along the developmental trajectory (Dye and Bavelier, 2010).

An animal study indicated that those improvements in visual performances were caused by cross-modal functional reorganization of auditory cortex in individuals with early or congenitally hearing loss, and several experimental approaches had been used to localize individual visual functions in discrete portions of reorganized auditory cortex (Lomber et al., 2010). Projections from a number of fields in the visual cortex were observed in the dorsal zone of the auditory cortex (Barone et al., 2013) and the posterior auditory field (Butler et al., 2017). Furthermore, the structural connectivity changes between visual and auditory cortices contribute to reduced resting-state alpha band activity in the posterior auditory field (Yusuf et al., 2017) and reduced stimulus-related effective connectivity from higher-order auditory cortical areas involved in the cross-modal reorganization to primary areas (Yusuf et al., 2021) in congenitally hearing loss cats.

Studies of hearing loss in human beings have revealed neural activity changes associated with cortical plasticity. Karns et al. (2012) found that adults with congenitally hearing loss exhibited a greater visual stimulating response in the Heschl’s gyrus compared with normal-hearing controls. Finney et al. (2001, 2003) also reported a visual-evoked activation of the auditory cortex in individuals with early hearing loss. Dewey and Hartley (2015) found that participants with profoundly hearing loss presented a larger group response elicited by visual motion within the right temporal lobe compared with normal-hearing controls.

Previous investigations have demonstrated cross-modal recruitment of auditory cortical areas in subjects with profoundly hearing loss viewing sign language (Nishimura et al., 1999; Petitto et al., 2000; MacSweeney et al., 2002). Visual cross-modal reorganization is dependent on hearing loss severity and is also observed in adults with mild-moderate hearing loss (Campbell and Sharma, 2014, 2020; Glick and Sharma, 2017). On one hand, this kind of cortical plasticity contributes to superior visual performance abilities and increased visual-evoked activation of the auditory cortex. On the other hand, investigators (Doucet et al., 2006; Sandmann et al., 2012; Lazard and Giraud, 2017) have also found that auditory–visual reorganization impacts rehabilitation of individuals with hearing loss after applying restorative strategies such as cochlear implantation (CI). In these studies, enhanced visual-evoked activation of the auditory cortex was always associated with poor speech comprehension in individuals with early or congenitally hearing loss following CI. As a result, it has been assumed that visual language is maladaptive for hearing restoration with a CI. However, a recent study has reported that visual stimulus provides adaptive benefits to speech processing following CI (Anderson et al., 2017). These inconsistent study results indicate that it is necessary to further investigate the cross-modal reorganization, especially brain causal connectivity network changes following auditory–visual reorganization and their effects on cognitive and speech processing abilities of hearing loss patients. Here, we study this issue. We hypothesize that cross-modal functional reorganization contributes to changes in the brain causal connectivity network, which may affect cognitive and speech processing abilities of patients with hearing loss. Auditory and visual language signals might have different effects on speech processing.

## Materials and Methods

### Participants

Written informed consent was obtained from all participants according to a protocol approved by the Institutional Ethics Committee of Shandong First Medical University. In the current study, the severity levels of the hearing loss are labeled according to the Global Burden of Disease (GBD) 2013 and World report on hearing (i.e., mild: 20 to <35 dB, moderate: 35–49 dB, moderately severe: 50–64 dB, severe: 65–79 dB, profound: 80–94 dB, and complete ≥ 95 dB) (Xu et al., 2019b; World Health Organization, 2021). The less severe hearing loss is used as evaluating criteria in the bilateral. Twelve patients with long-term bilateral mild-to-severe sensorineural hearing loss and 12 age-, sex-, and education-matched normally hearing volunteers participated in this study. Hearing loss participants were asked about their hearing loss, including the etiology of hearing loss, age at onset and duration of hearing loss, duration of tinnitus, and hearing aid experience. Only one participant had a year of hearing aid use, and others had no hearing aid experience. All patients had post-lingual hearing loss, and 11 of them had self-reported tinnitus with a mean duration of 14.4 years ± 5.9 (standard error), ranging from 10 days to 55 years. Hearing loss was acquired due to infection in two patients and to noise and traumatism in one patient, and hearing loss in others had an unknown cause. All participants were right-handed and had normal or corrected-to-normal vision. None of them had a history of medical and stroke/cerebrovascular ischemia.

**FIGURE 1:**
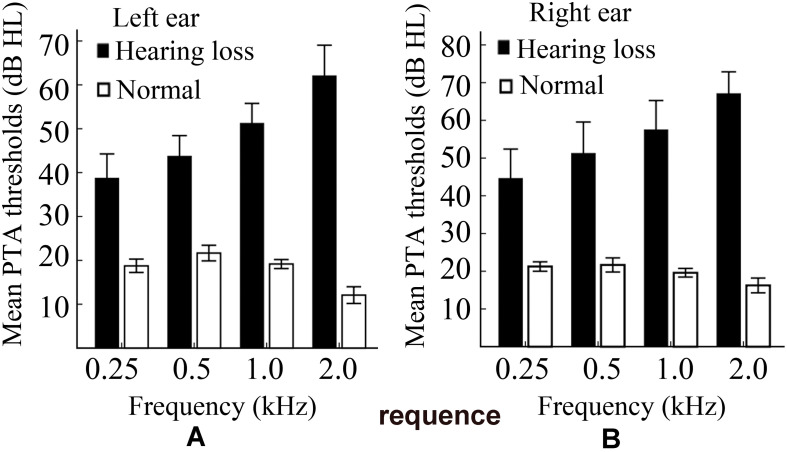
A plot of the non-aided audiograms. **(A)** The non-aided audiogram of the left ear. **(B)** The non-aided audiogram of the right ear. PTA, pure tone audiometry; HL, hearing level.

A clinical audiologist performed pure tone audiometry (PTA) using an Astera audiometer in the Department of Otorhinolaryngology and Head-neck Surgery of the Second Affiliated Hospital of Shandong First Medical University. Hearing level (HL) was defined as a speech-frequency pure tone average of air conduction thresholds at 0.25, 0.5, 1.0, and 2 kHz. All of the normally hearing participants had a HL of less than 27 dB, while all of the hearing loss patients had a HL of more than 30 dB. Non-aided audiograms based on pure-tone audiometry for these participants are shown in Figure 1. Details of the demographic characteristics of participants are shown in Table 1. Data of 21 subjects had been from a previously published study (Xu et al., 2017).

**TABLE 1:** Demographic characteristics of participants.

Characteristics	Hearing loss (*n* = 12)	Normal (*n* = 12)	Statistic (df)	*p*-value
Age (yrs)	55.7 ± 2.0	52.3 ± 1.8	*T* = 1.256 (22)	0.222
Education (yrs)	10.1 ± 0.7	11.7 ± 0.9	*T* = −1.373 (22)	0.184
Male/Female	9/3	5/7	χ^2^ = 2.743 (1)	0.098
Threshold (dB HL)	L: 49.0 ± 4.1	L: 17.6 ± 1.3	*T* = 7.229 (13.2)	<0.001*
	R: 55.1 ± 7.1	R: 19.7 ± 1.3	*T* = 4.925 (11.8) **	<0.001*
	L: 49.0 ± 4.1 R: 55.1 ± 7.1	–	*T* = -0.751 (22)^#^	0.461
	–	L: 17.6 ± 1.3 R: 19.7 ± 1.3	*T* = -1.094 (22)^#^	0.286
Hearing loss duration (yrs)	16.7 ± 4.5	na	–	–

Data are mean ± standard errors. yrs, years; L, left; R, right; df, degrees of freedom; na, not applicable; HL, hearing level.

*p < 0.05 is considered to indicate a significant difference.

**Homogeneity test (Levene’s test), *p* < 0.05, revised *t*-test.

*^#^Comparisons of hearing levels across both left and right ears (two-sample *t*-tests). Age of the hearing loss participant ranges from 39 to 63 years; onset of hearing loss ranges from 5 to 58 years of age; the duration of hearing loss ranges from 3 to 55 years; hearing level of left ear ranges from 31.25 to 76.25 dB HL; hearing level of right ear ranges from 35 to 115 dB HL; age of the normally hearing volunteer ranges from 42 to 63 years; hearing level of left ear ranges from 11.25 to 26.25 dB HL; hearing level of right ear ranges from 8.75 to 26.25 dB HL.*

Participants were excluded if their head movements were greater than 2.5 mm or 2.5° during imaging. No participants were excluded in the present study.

### Neuropsychological Tests

Mini-mental state examination (MMSE) is sensitive for measuring cognitive impairment (Tombaugh and Mcintyre, 1992). In this study, MMSE and Wechsler adult intelligence test (Wechsler, 1997) were performed to assess the cognitive abilities of the participants. Full details of all tests had been published in a previous study (Zhang et al., 2015).

It has been demonstrated that hearing impairment negatively impacts cognitive test performance (Füllgrabe, 2020a). To minimize the influence of hearing loss on the assessment of cognitive performances. All neuropsychological tests were administered in a quiet setting with minimal distractions and were provided in Mandarin. An experienced examiner had been accustomed to working with hearing loss patients and verbally gave all instructions in a face-to-face manner. However, as none of these precautions offset a possible bias introduced by comparing normal-hearing and hearing-impaired participants on an auditory cognitive task, the reported cognitive data are possibly biased and their association with changes in brain connectivity needs to be interpreted with caution (Füllgrabe, 2020b). Participants were tested on (a) MMSE, (b) digit symbol substitution test (DSST), (c) forward digit spans (FDS) and backward digit spans (BDS), (d) verbal fluency (VF), (e) trail making test part A (TMTA), (f) trail making test part B (TMTB), (g) Stroop color–word test A (SCWA), (h) Stroop color–word test B (SCWB), and (i) Stroop color–word test C (SCWC).

### Magnetic Resonance Imaging (MRI) Data Acquisition

We acquired MRI data of 24 participants using a GE Discovery MR 750 3-T scanner. MRI data of 21 participants had been from a previously published study (Xu et al., 2017). Functional images were collected axially using an echo-planar imaging sequence (echo time: 30 ms; repetition time: 2000 ms; 41 slices, 64 × 64 matrix; voxel size: 3.4375 mm × 3.4375 mm × 3.2 mm). T1-weighted structural images were also acquired with parameters: echo time = 8.156 ms; repetition time = 3.18 ms; 176 slices with 1 mm × 1 mm × 1 mm voxels.

All of the participants underwent a functional magnetic resonance scan during a conscious resting state with their eyes closed. To reduce noise and disturbance, these participants wore earplugs and headphones and were instructed to keep still, as motionless as possible and not to think about anything in particular. Head movement was minimized by placing soft pads at the sides of the head. The light was switched off during the resting-state scanning.

### Data Preprocessing

All of the MRI data were firstly preprocessed by using the software package spm8^1^. Slice timing, motion correction, space normalization, and spatial smoothing (8 mm × 8 mm × 8 mm) were first executed. Segmentation for structural images generated white matter, gray matter, and cerebrospinal fluid images. Full details of all preprocessing steps are provided in the previously published study (Xu et al., 2017). Then, we performed the following procedures utilizing the virtual digital brain software package VDB1.6^2^ for the preprocessed data: (1) the removal of linear and quadratic trends; (2) regressing out covariates including realignment parameters, mean white matter, and cerebrospinal fluid signals; (3) band-pass temporal filtering (0.01–0.1 Hz); and (4) calculating the interregional causal connections of each participant. Finally, we obtained the nodal degrees and the shortest causal connectivity paths.

### The Shortest Causal Connectivity Paths and Transfer Probability

In the current study, causal connectivity was realized by entropy connectivity method, as defined in a previous study (Zhang et al., 2016), and the causal connectivity is related to BOLD (blood oxygen level-dependent) signal amplitude. Suppose there is a strong correlation between amplitude changes of the BOLD signal across two brain regions; moreover, the current change of the BOLD signal amplitude in one brain region presents synchronous or asynchronous coupling with the future change of the other, then there exist a causal connectivity between the two brain regions (i.e., one is response system, and the other is drive system).

**FIGURE 2:**
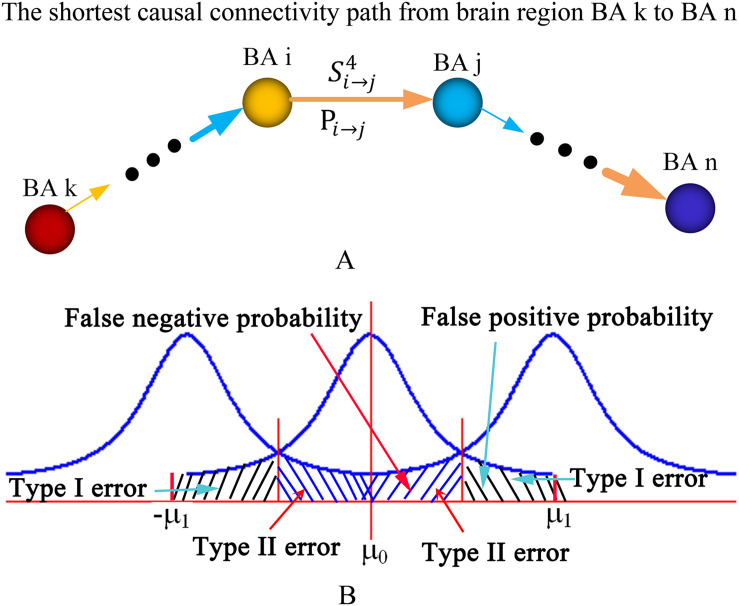
Diagram of the shortest causal connectivity path, transfer probability, and type I and II errors. **(A)** The diagram of the shortest causal connectivity path and transfer probability. **(B)** The diagram of type I error and type II error. The curves in the diagram are statistical distributions. The parameters *μ*_0_ and *μ*_1_ are the means or expectations of the distributions. The value of *μ*_0_ is equal to zero, and the value of *μ*_1_ depends on the actual appilcation. BA, Brodmann’s Area.

Let, L_*k →n*_ denote the length of the shortest causal connectivity path from brain region BA *k* to *n*, then L_*k →n*_ was defined as:

(1) $$L_{k\to n}=\sum 1/S_{i\to j}^{4}$$

where, *S*_*i →j*_ denotes the strength of causal connectivity from brain region BA *i* to *j*. BA *i* and BA *j* are two adjacent brain regions in the shortest causal connectivity path from BA *k* to *n* (Figure 2A). The shortest causal connectivity path reflects the strongest causal interaction from one brain region to the other. Let *TP*_*k*→*n*_ denote the transfer probability of the shortest causal connectivity path from brain region BA *k* to *n*, then *TP*_*k*→*n*_ was defined as:

(2) $$T\,P_{k\to n}=\prod P_{i\to j}$$

where, *P*_*i →j*_ denotes the probability of synchronous or asynchronous causal connectivity from brain region BA *i* to *j* (Figure 2A). The transfer probability reflects the influence extent of one brain region’s neural activity on the other through the shortest causal connectivity path. *TP*_*k*→*n*_ > 0 indicates a positive influence, and *TP*_*k*→*n*_ < 0 indicates a negative influence. In general, *TP*_*k*→*n*_ > 0.05 or *TP*_*k*→*n*_ < −0.05 is regarded as a significant influence.

### Positive Reproducible Test (PRT)

In statistical hypothesis testing, a type I error is the incorrect rejection of a true null hypothesis (i.e., a “false positive”), while a type II error is the failure to reject a false null hypothesis (i.e., a “false negative”). A false positive probability is regarded as the exact probability of committing a type I error or the exact level of significance (i.e., *p*-value). A false-negative probability is the exact probability of committing a type II error (Figure 2B). Types I and II error probabilities (i.e., false-positive and -negative probabilities) are a zero-sum game for any given sample size. Any method that protects more against one type of error is guaranteed to increase the probability of the other kind of error (Lieberman and Cunningham, 2009). Reduced type I error will lead to increased type II error. Thus, we suppose a positive reproducible test method to obtain a trade-off between the false-positive and -negative probabilities. This method is described as follows: (1) set *p*-value (i.e., false-positive probability) as a significant level in a statistical hypothesis testing for all samples and obtain *n* positive effects *A_1_*,*A*_2_, ⋯, *A_n_*; (2) randomly select *k* samples and repeat the statistical hypothesis testing; (3) repeat step (2) *M* times; (4) if the positive effect *A_i_* occurs *m_i_* times, then the reproducible rate *R_i_* of the positive effect *A_i_* is defined as

(3) $$R_{i}=\frac{m_{i}}{M}$$

where, *i*1,2,⋯,*n*, higher *R_i_* means fewer type I errors. This supposed method can obtain low false-negative probability and few type I errors through selecting high *R_i_* in the statistical hypothesis testing.

### Causal Connections and Nodal Degrees

The total number of synchronous or asynchronous input causal connectivity of a brain region is defined as synchronous or asynchronous input nodal degree of the brain region. Similarly, the total number of synchronous or asynchronous output causal connectivity of a brain region is defined as synchronous or asynchronous output nodal degree of the brain region.

Enhanced synchronous input causal connectivity and increased synchronous input nodal degree of a brain region mean that some original functions associated with the brain region will be weakened due to increased resource occupation. Similarly, enhanced asynchronous input causal connectivity and increased asynchronous input nodal degree of a brain region indicate that neural activity of the brain region will be depressed and related functions will be weakened.

Weakened synchronous input causal connectivity and reduced synchronous input nodal degree of a brain region imply that some functions associated with the brain region will be enhanced due to decreased resource occupation. Similarly, weakened asynchronous input causal connectivity and reduced asynchronous input nodal degree of a brain region indicate that the neural activity of this brain region will be enhanced due to reduced neural inhibition.

Enhanced synchronous output causal connectivity and increased synchronous output nodal degree of a brain region imply that the information processing ability of the brain region will be enhanced due to occupying more resources. Similarly, enhanced asynchronous output causal connectivity and increased asynchronous output nodal degree of a brain region indicate that the brain region enhances its information processing abilities through depressing neural activities of other brain regions.

Weakened synchronous output causal connectivity and reduced synchronous output nodal degree of a brain region imply that the information processing ability associated with the brain region will decline due to interrupted causal connectivity. Similarly, weakened asynchronous output causal connectivity and reduced asynchronous output nodal degree of a brain region imply the decline of some functions associated with the brain region.

All performances were executed using the VDB1.6 software package.

### Statistical Analyses

We performed two-sample *t*-tests to examine group differences in age, educational levels, hearing levels, and neuropsychological test scores. A Chi-square test was conducted to examine group differences in sex. Statistical analyses were conducted using SPSS software (version 19.0), and a *p* < 0.05 was considered to indicate a significant difference in any single analysis. A *p* < 0.05 (FDR corrected) is considered as a significant difference across the hearing loss and normally hearing control group for the two-sample *t*-tests of neuropsychological performance scores.

## Results

### Neuropsychological Test Results

The results of neuropsychological tests are summarized in Table 2. Compared with normally hearing controls, patients with hearing loss presented significantly decreased test scores (*p* < 0.05, FDR corrected) in MMSE, VF, and BDS, but significantly increased test scores (*p* < 0.05, FDR corrected) in TMTA, SCWTA, SCWTB, and SCWTC. A lower score means a worse performance for MMSE, VF, and BDS. A higher score means a worse performance for TMTA, SCWTA, SCWTB, and SCWTC. No significant difference was observed in other tests across the two groups.

**TABLE 2:** Summary of neuropsychological tests.

Tests	Hearing loss (*n* = 12)	Normal (*n* = 12)	Statistic (df)	*p*-value
MMSE	27.3 ± 0.54	29 ± 0.30	*T* = −2.776 (17.3)**	0.013*
DSST	35.4 ± 4.1	45.1 ± 3.8	*T* = −1.735 (22)	0.370
VF	25.7 ± 1.8	32.8 ± 1.8	*T* = −2.807 (22)	0.01*
FDS	6.9 ± 0.29	7.6 ± 0.26	*T* = −1.72 (22)	0.099
BDS	4.2 ± 0.17	5.2 ± 0.34	*T* = −2.613 (22)	0.016*
TMTA	58.6 ± 5.62	42.7 ± 2.47	*T* = 2.589 (15.1)**	0.020*
TMTB	160.6 ± 16.5	123.0 ± 11.3	*T* = 1.876 (22)	0.074
SCWA	30.5 ± 2.1	24.3 ± 1.3	*T* = 2.519 (18.4)**	0.021*
SCWB	47.7 ± 3.8	36.3 ± 1.9	*T* = 2.677 (22)	0.014*
SCWC	86.3 ± 6.14	65.5 ± 4.7	*T* = 2.683 (22)	0.014*

Data are mean ± standard errors. df, degrees of freedom.

*p < 0.05 (FDR corrected) is considered as a significant difference.

**Homogeneity test (Levene’s test), *p* < 0.05, revised *t*-test. MMSE, mini-mental state examination; DSST, digit symbol substitution test; VF, verbal fluency; FDS, forward digit spans; BDS, backward digit spans; TMTA, trail making test part A; TMTB, trail making test part B; SCWTA, Stroop color–word test A; SCWTB, Stroop color–word test B; SCWTC, Stroop color–word test C.

**TABLE 3:** Indexes and corresponding brain regions.

Indexes	Brain regions	Indexes	Brain regions
BA 2L	Left primary somatosensory cortex	BA 11R	Right orbitofrontal cortex
BA 4L	Left primary motor cortex	BA 13L	Left insular cortex
BA 5L	Left somatosensory association cortex	BA 13R	Right insular cortex
BA 5R	Right somatosensory association cortex	BA 17R	Right primary visual cortex
BA 8R	Right dorsal frontal cortex	BA 18R	Right secondary visual cortex
BA 9R	Right dorsolateral prefrontal cortex	BA 19R	Right associative visual cortex
BA 9L	Left dorsolateral prefrontal cortex	BA 19L	Left associative visual cortex
BA 24R	Right ventral anterior cingulate cortex	BA 25L	Left subgenual cortex
BA 32R	Right dorsal anterior cingulate cortex	BA 30L	Left cingulate cortex
BA 34L	Left anterior entorhinal cortex	BA 36L	Left parahippocampal cortex
BA 36R	Right parahippocampal cortex	BA 38R	Right temporopolar area
BA 38L	Left temporopolar area	BA 40L	Left supramarginal gyrus
BA 41L	left primary auditory cortex	BA 41R	Right primary auditory cortex
BA 42L	left primary auditory cortex	BA 43L	Left subcentral area
BA 46R	Right dorsolateral prefrontal cortex	BA 44R	Right IFC pars opercularis
BA 46L	Left dorsolateral prefrontal cortex	BA 45L	Left IFC pars triangularis

### Changes of Causal Connections

We studied changes of causal connections and nodal degrees in patients with hearing loss, as well as the relationships between nodal degrees and cognitive performance scores. The strength of interregional causal connectivity was corrected using the positive reproducible test (PRT) method. Only those significant changes and correlations are described as follows.

**FIGURE 3:**
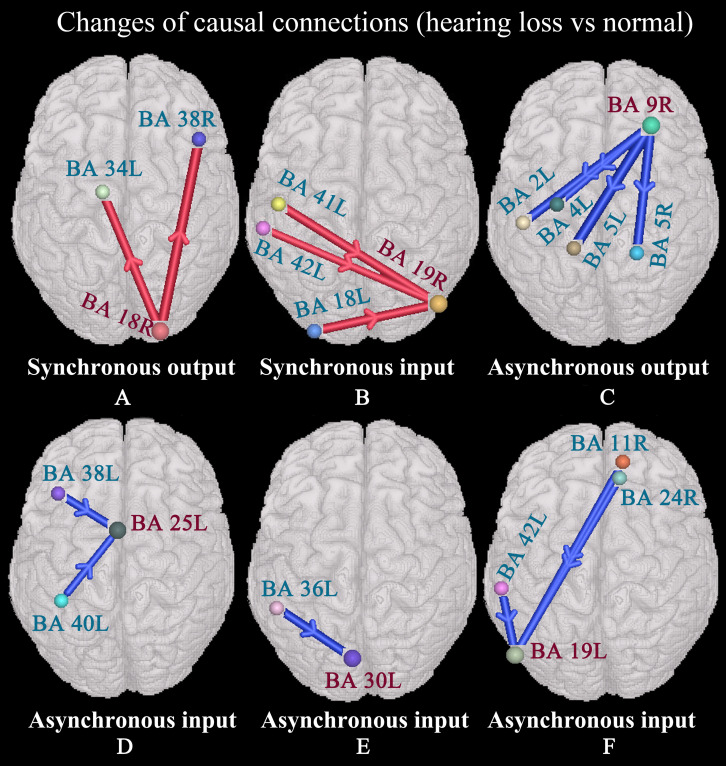
Changes of causal connections in patients with hearing loss (*p* < 0.05, two-sided, PRT corrected; reproducible rate: 0.5; number of subjects: 10; PRT threshold: 4000). The red bar denotes enhanced causal connectivity, and the blue bar denotes weakened causal connectivity. The width of the bar indicates the strength of causal connectivity. The direction of the arrow indicates the direction of causal connectivity. See Table 3 for the BA index. **(A)** Enhanced synchronous output causal connections for BA 18R. **(B)** Enhanced synchronous input causal connections for BA 19R. **(C)** Weakened asynchronous output causal connections for BA 9R. **(D)** Weakened asynchronous input causal connections for BA 25L. **(E)** Weakened asynchronous input causal connections for BA 30L. **(F)** Weakened asynchronous input causal connections for BA 19L.

We found that the right secondary visual cortex (BA 18R) presented enhanced synchronous output causal connections with the left anterior entorhinal cortex (BA 34L) and right temporopolar area (BA 38R) (Figure 3A); the right associative visual cortex (BA 19R) exhibited enhanced synchronous input causal connectivity with the left primary auditory cortex (BAs 41L and 42L) and secondary visual cortex (BA 18L) (Figure 3B); the right dorsolateral prefrontal cortex (BA 9R) exhibited weakened asynchronous output causal connections with the left primary somatosensory cortex (BA 2L), left primary motor cortex (BA 4L), and the somatosensory association cortex (BAs 5L and 5R) (Figure 3C); the left subgenual cortex (BA 25L) presented weakened asynchronous input causal connectivity with the left temporopolar area (BA 38L) and supramarginal gyrus (BA 40L) (Figure 3D); the left cingulate cortex (BA 30L) presented weakened asynchronous input causal connections with the left parahippocampal cortex (BA 36L) (Figure 3E); the left associative visual cortex (BA 19L) presented weakened asynchronous input causal connectivity with the left primary auditory cortex (BA 42L), right orbitofrontal cortex (BA 11R), and the ventral anterior cingulate cortex (BA 24R) (Figure 3F).

### Changes of Nodal Degrees

**FIGURE 4:**
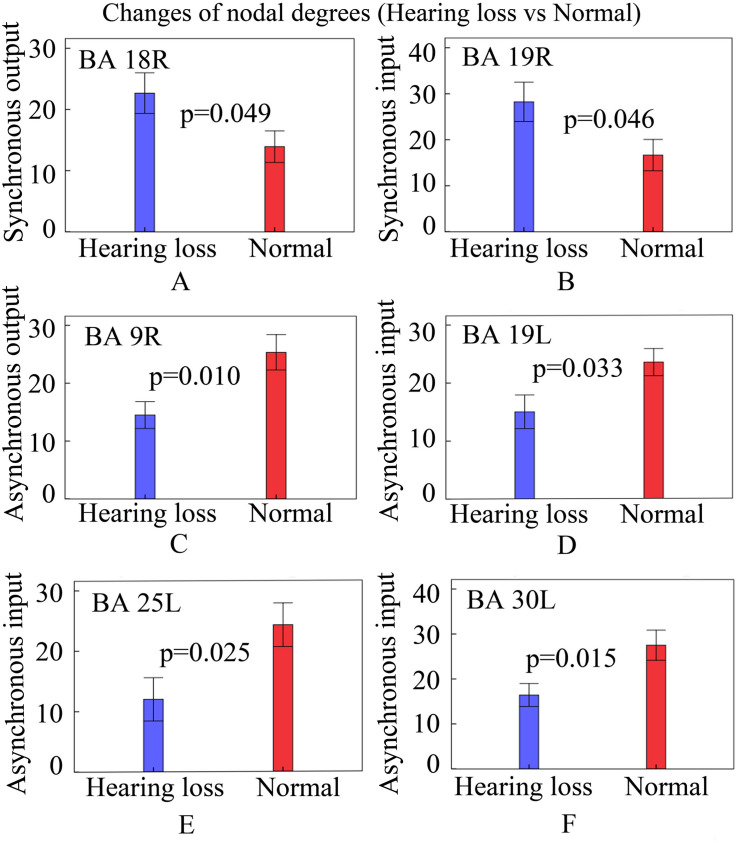
Changes of nodal degrees in patients with hearing loss. Bars: mean ± standard errors. See Table 3 for the BA index. **(A)** The bar graph of synchronous output nodal degrees for BA 18R. **(B)** The bar graph of synchronous input nodal degrees for BA 19R. **(C)** The bar graph of asynchronous output nodal degrees for BA 9R. **(D)** The bar graph of asynchronous input nodal degrees for BA 19L. **(E)** The bar graph of asynchronous input nodal degrees for BA 25L. **(F)** The bar graph of asynchronous input nodal degrees for BA 30L.

In order to observe whether causal connectivity changes had an impact on nodal degrees, we studied the nodal degree utilizing two-sample *t*-tests and found significant changes (*p* < 0.05, two-sided) in hearing loss patients. The results are shown in Figure 4.

Compared with normally hearing subjects, patients with hearing loss presented increased synchronous output nodal degrees in BA 18R (Figure 4A); increased synchronous input nodal degrees in BA 19R (Figure 4B); reduced asynchronous output nodal degrees in BA 9R (Figure 4C); and reduced asynchronous input nodal degrees in BAs 19L, 25L, and 30L (Figures 4D–F).

### Changes of the Shortest Causal Connectivity Paths

**FIGURE 5:**
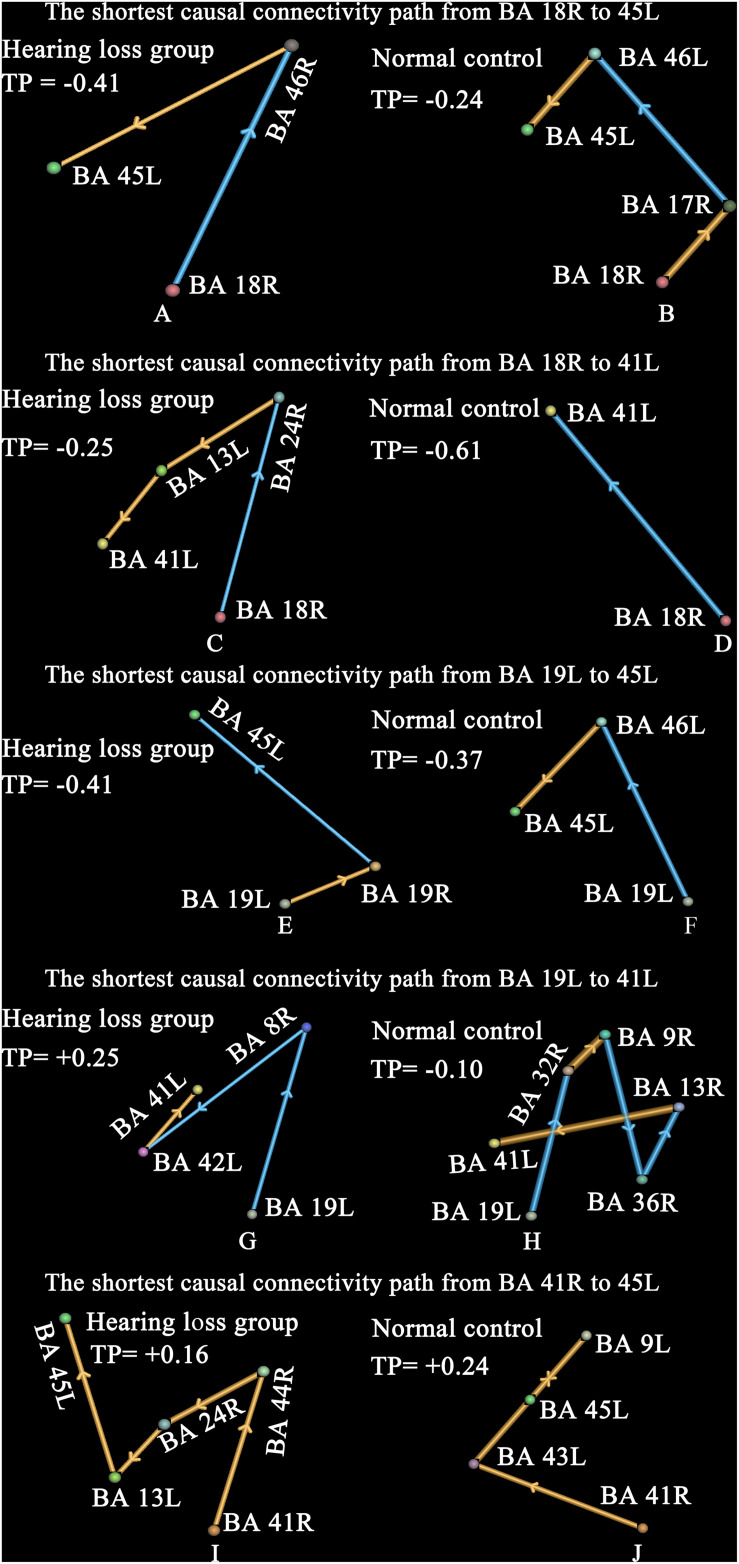
The shortest causal connectivity paths and transfer probabilities. Strengths of interregional causal connections were corrected by using positive reproducible test (PRT) method: *p* < 0.05, PRT corrected; reproducible rate 0.5; number of subjects 10; PRT threshold 1000. The golden bar denotes synchronous causal connectivity, and the light blue bar denotes asynchronous causal connectivity. The width of the bar indicates the strength of causal connectivity. The direction of the bar indicates the direction of causal connectivity. The transfer probability indicates the influence of one brain region on the other through the shortest causal connectivity path. Transfer probability > 0.05 denotes a significant positive effect, and transfer probability < −0.05 denotes a significant negative effect. See Table 3 for the BA index. **(A,B)** Denote the shortest causal connectivity path from BA 18R to 45L in the hearing loss and the normal control, respectively. **(C,D)** Denote the shortest causal connectivity path from BA 18R to 41L in the hearing loss and the normal control, respectively. **(E,F)** Denote the shortest causal connectivity path from BA 19L to 45L in the hearing loss and the normal control, respectively. **(G,H)** Denote the shortest causal connectivity path from BA 19L to 41L in the hearing loss and the normal control, respectively. **(I,J)** Denote the shortest causal connectivity path from BA 41R to 45L in the hearing loss and the normal control, respectively.

The shortest causal connectivity path from one brain region to the other reflects the strongest causal interaction between these two brain regions. It is unclear whether the change of nodal degree also leads to the change of the shortest causal connectivity path; we studied this issue. Our study focused on the shortest causal connectivity paths between the visual cortex, auditory cortex, and those brain areas associated with speech processing. The results are shown in Figure 5.

Compared with normal-hearing subjects, patients with hearing loss presented a shorter causal connectivity path (*p* < 0.0001, two-sided, two-sample *t*-test, uncorrected) and a bigger transfer probability (hearing loss group = −0.41, normal-hearing control = −0.24) from BA 18R to Broca’s area (BA 45L) (Figures 5A,B). In addition, we also studied the shortest causal connectivity paths and transfer probabilities (hearing loss group = −0.25, normal-hearing control = −0.61) from BA 18R to 41L (Figures 5C,D); BA 19L to 45L with transfer probabilities (hearing loss group = −0.41, normal-hearing control = −0.37) (Figures 5E,F); BA 19L to 41L with transfer probabilities (hearing loss group = +0.25, normal-hearing control = −0.10) (Figures 5G,H); and BA 41R to 45L with transfer probabilities (hearing loss group = +0.16, normal-hearing control = +0.24) (Figures 4I,J). The transfer probability > 0.05 or < −0.05 indicates that the change of neural signal in one brain region has significant effects on the other through the shortest causal connectivity path from one brain region to the other.

### Correlation Analysis

**FIGURE 6:**
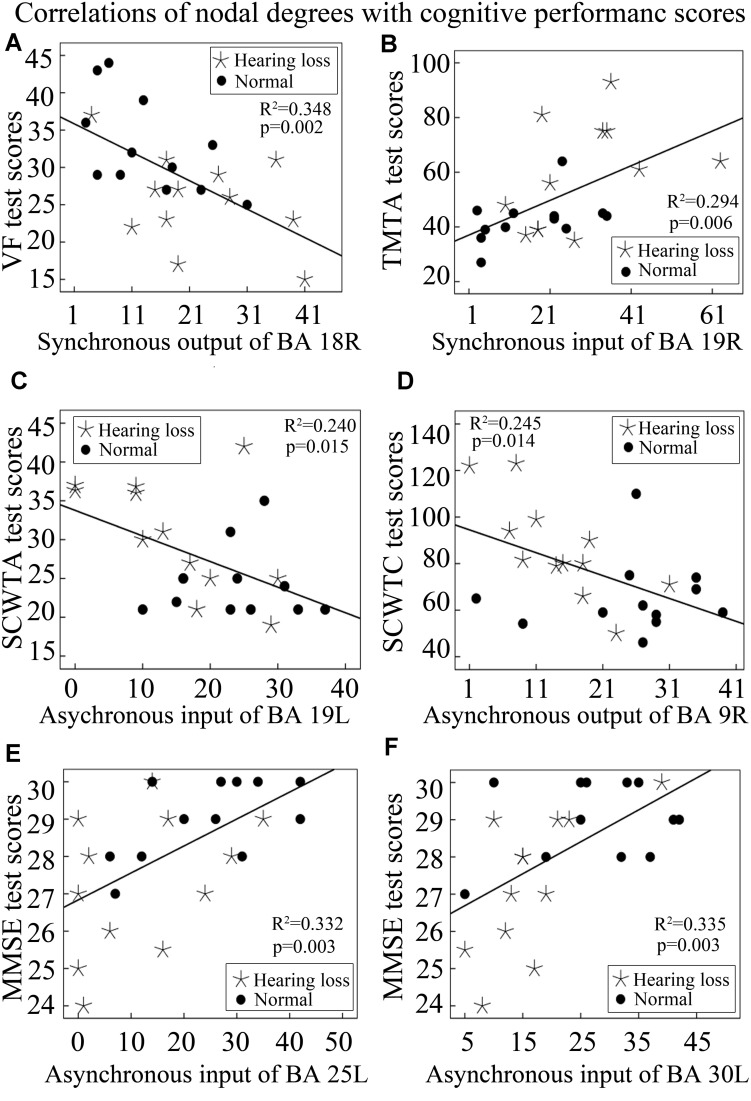
Correlations between nodal degrees and cognitive performance scores. See Table 3 for the BA index. **(A)** The correlation of synchronous output nodal degrees for BA 18R with VF test scores. **(B)** The correlation of synchronous input nodal degrees for BA 19R with TMTA test scores. **(C)** The correlation of asynchronous input nodal degrees for BA 19L with SCWTA test scores. **(D)** The correlation of asynchronous output nodal degrees for BA 9R with SCWTC test scores. **(E)** The correlation of asynchronous input nodal degrees for BA 25L with MMSE test scores. **(F)** The correlation of asynchronous input nodal degrees for BA 30L with MMSE test scores.

In order to investigate whether nodal degree changes can contribute to cognitive decline, we studied the relationship of cognitive performance scores with nodal degrees utilizing Pearson’s correlation analysis method. The results are described as follows. VF test scores exhibited a significant negative correlation with synchronous output nodal degrees of BA 18R (Figure 6A); TMTA test scores exhibited a significant positive correlation with synchronous input nodal degrees of BA 19R (Figure 6B); SCWTA test scores presented a significant negative correlation with asynchronous input nodal degrees of BA 19L (Figure 6C); SCWTC test scores presented a significant negative correlation with asynchronous output nodal degrees of BA 9R (Figure 6D); MMSE test scores presented significant positive correlations with asynchronous input nodal degrees of both BA 25L and BA 30L (Figures 6E,F).

**FIGURE 7:**
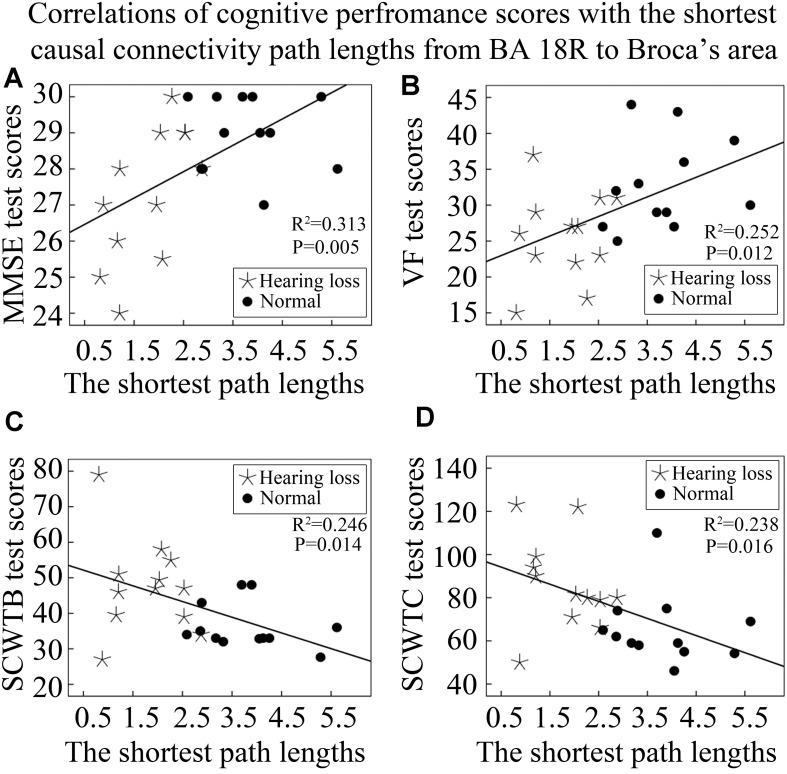
Correlations between cognitive performance scores and lengths of the shortest causal connectivity paths from BA 18R to Broca’s area (BA 45L). See Table 3 for the BA index. **(A)** The correlation of MMSE test scores with the shortest path lengths from BA 18R to Broca’s area. **(B)** The correlation of VF test scores with the shortest path lengths from BA 18R to Broca’s area. **(C)** The correlation of SCWTB test scores with the shortest path lengths from BA 18R to Broca’s area. **(D)** The correlation of SCWTC test scores with the shortest path lengths from BA 18R to Broca’s area.

In addition, we also analyzed the correlations of cognitive performance scores with lengths of the shortest causal connectivity paths from BA 18R to Broca’s area (BA 45L). We found that both MMSE and VF test scores were positively correlated with lengths of the shortest causal connectivity paths from BA 18R to BA 45L (Figures 7A,B). On the contrary, SCWTB and SCWTC test scores presented negative correlations with the path lengths (Figures 7C,D).

**FIGURE 8:**
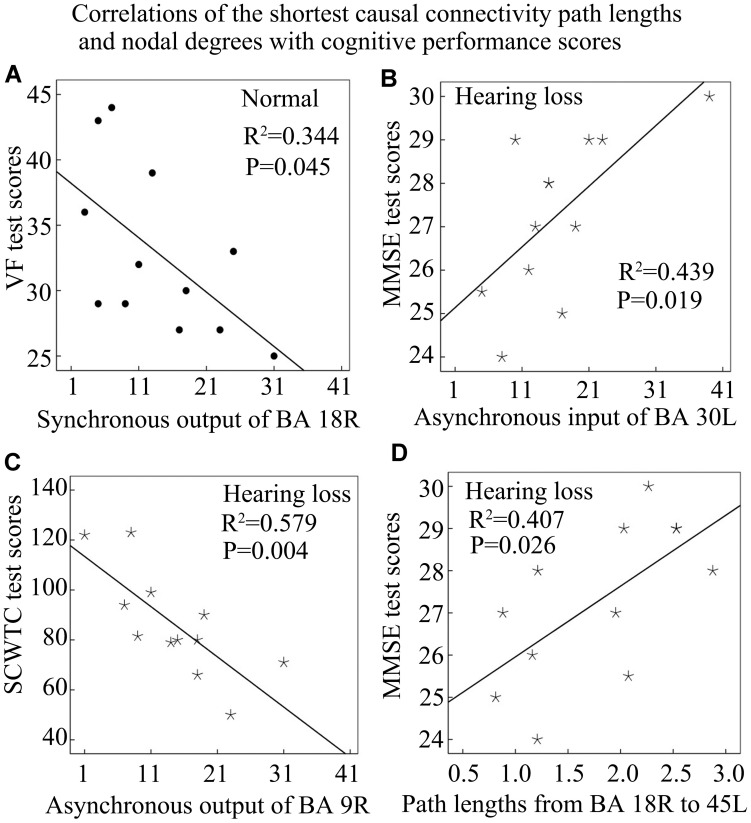
Correlations of the shortest causal connectivity path lengths and nodal degrees with cognitive performance scores in the control or the hearing loss group. See Table 3 for the BA index. **(A)** The correlation of synchronous output nodal degrees for BA 18R with VF test scores in the normal control group. **(B)** The correlation of asynchronous input nodal degrees for BA 30L with MMSE test scores in the hearing loss group. **(C)** The correlation of asynchronous output nodal degrees for BA 9R with SCWTC test scores in the hearing loss group. **(D)** The correlation of the shortest path lengths from BA 18R to 45L with MMSE test scores in the hearing loss group.

In this study, we have mixed controls and hearing loss subjects into one set and computed correlations (Figures 6, 7). This may lead to a curious correlation given the fact that the hearing loss subjects may simply show a different outcome than controls. To verify this issue, we recomputed those correlations by separating controls and hearing loss subjects and found that only several correlations are significant: synchronous output nodal degrees of BA 18R with VF test scores in the control group (Figure 8A); MMSE test scores with asynchronous input nodal degrees of BA 30L in the hearing loss group (Figure 8B); SCWTC test scores with asynchronous output nodal degrees of BA 9R in the hearing loss group (Figure 8C); and lengths of the shortest causal connectivity paths from BA 18R to BA 45L with MMSE test scores in the hearing loss group (Figure 8D).

**FIGURE 9:**
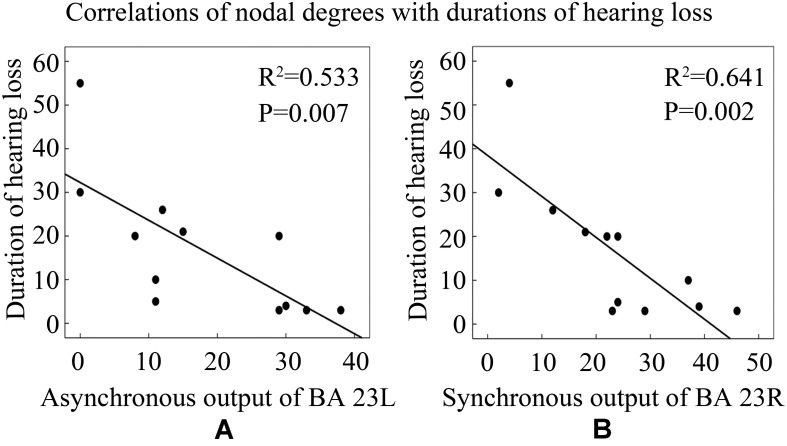
Correlations between nodal degrees and durations of hearing loss. See Table 3 for the BA index. **(A)** The correlation of asynchronous output nodal degrees for BA 23L with durations of hearing loss (years). **(B)** The correlation of synchronous output nodal degrees for BA 23R with durations of hearing loss (years).

To further explore the influence of hearing loss on causal connectivity network, we also studied the relationships between nodal degrees and durations of hearing loss. Pearson’s correlation analysis revealed that the asynchronous output nodal degree of BA 23L strongly negatively related with the duration of hearing loss (Figure 9A); similarly, the synchronous output nodal degree of BA 23R strongly negatively related with the duration of hearing loss as well (Figure 9B).

## Discussion

The present study investigated adult-onset hearing loss and its effects on the connectivity in the brain using resting-state functional magnetic resonance imaging (fMRI). The main findings of the study are described as follows. Patients with hearing loss presented increased synchronous input causal connectivity for the right associative visual cortex with the auditory cortex and exhibited a shorter causal connectivity path from the right secondary visual cortex to Broca’s area. These findings were consistent with cross-modal reorganization. Moreover, we also observed nodal degree changes in the right secondary visual cortex, associative visual cortex, right dorsolateral prefrontal cortex, left subgenual cortex, and the left cingulate cortex. These changes were significantly related with poor cognitive performances. In addition, we also noted that changes of neural signal in the visual cortex had negative effects on neural activity of Broca’s area but, in the auditory cortex, had positive effects on neural activity of Broca’s area through the shortest causal connectivity paths.

### Auditory–Visual Inhibition and Cross-Modal Reorganization

Molloy et al. (2015) found that a visual search task of high perceptual load led to reduced auditory evoked activity in auditory cortical areas compared with that of low perceptual load. Moreover, the reduction in neural responses of the auditory cortex was associated with reduced awareness of the sound. Study on animals found that activation of the auditory cortex by a noise burst suppressed neural activities of the visual cortex *via* cortico-cortical inhibitory circuits (Iurilli et al., 2012). These findings support a neural account of shared audiovisual resources, which lead to auditory–visual inhibition. Our results are consistent with previous studies. Furthermore, this current study also found that patients with hearing loss presented reduced auditory–visual inhibition through the shortest causal connectivity path from the right secondary visual cortex to the left primary auditory cortex (BA 41L) and weakened asynchronous input causal connectivity from the left primary auditory cortex (BA 42L) to the left associative visual cortex. Reduced auditory–visual inhibition might contribute to the auditory cortex recruited by visual modality. In addition, hearing loss patients also exhibited enhanced synchronous input causal connectivity from the left primary auditory cortex to the right associative visual cortex, as well as enhanced causal connectivity through a shortest causal connectivity path from the left associative visual cortex to the left primary auditory cortex. These results suggest that there exists auditory–visual reorganization in patients with long-term bilateral mild-to-severe hearing loss.

### Influence of Visual and Auditory Signals on Neural Activities of Broca’s Area

Previous investigations found that enhanced visual-evoked activation in auditory brain regions of individuals with early or profoundly hearing loss was associated with poor speech comprehension (Doucet et al., 2006; Sandmann et al., 2012; Lazard and Giraud, 2017), and greater activation of auditory cortical areas in adult CI users during lip-reading predicted poorer speech understanding abilities (Strelnikov et al., 2013). Activation of auditory cortex by visual language might limit its capacity for auditory signal processing (Schormans et al., 2017). Therefore, auditory–visual reorganization is maladaptive for hearing restoration with a CI (Sandmann et al., 2012). It is well known that Broca’s area is associated with speech processing (Grewe et al., 2005; DeWitt and Rauschecker, 2012). This current study found that the signal from the visual cortex had a negative effect on neural activity of Broca’s area through the shortest causal interactive path with big transfer probability. In particular, the path from the visual cortex to Broca’s area is shorter in patients with hearing loss than in normally hearing controls; one possible reason is that hearing loss leads to auditory–visual reorganization, which contributes to larger synchronous output nodal degree of the visual cortex in hearing loss patients. As a result, more brain regions are linked with the visual cortex and lead to the shorter causal connectivity path, and these changes contribute to stronger interregional causal interaction and inhibition of visual signal for speech processing. Furthermore, shorter causal interactive paths from the visual cortex to Broca’s area and larger synchronous output nodal degrees of the visual cortex are associated with poorer speech processing performances manifested as lower VF test scores and higher SCWTB and SCWTC test scores in hearing loss patients. Measures of VF are mainly used to investigate language deficits (Tombaugh et al., 1999). Low VF test scores suggest poor speech processing. SCWTB and SCWTC performances are widely used to measure the ability of inhibiting cognitive interference (Scarpina and Tagini, 2017). High SCWTB and SCWTC test scores reflect a strong inhibition of the visual signal for speech processing. In addition, BA 18R in patients with hearing loss presented enhanced synchronous output casual connectivity with the left anterior entorhinal cortex (BA 34L) and right temporopolar area (BA 38R). BA 34L is associated with cognitive processing (Kovari et al., 2003), and BA 38R is involved in language processing and visual object naming (Poch et al., 2016). These results imply that visual language may be maladaptive for speech processing.

This current study found that the neural signal from the right primary auditory cortex (BA 41R) enhanced neural activity of Broca’s area through the shortest causal connectivity path with big transfer probability. Auditory language is adaptive for speech processing; thus current findings support the opinion that intensive and rigorous oral language training can improve language performance of patients with hearing loss following restorative strategies (Kos et al., 2009).

### Cognitive Decline Associated With Cerebral Cortex Plasticity

Behavior studies found that hearing loss was associated with incident all-cause dementia (Lin et al., 2011b) and poor cognitive performances such as low scores on DDST and MMSE tests (Lin, 2011; Xu et al., 2017), as well as memory and executive function tests (Lin et al., 2011a). A lot of adults over the age of 60 have both hearing loss and cognitive decline simultaneously (Lin et al., 2011c, 2013). Accumulating evidence from the clinical investigations indicates that there exists a link between hearing loss and cognitive decline (Lindenberger and Baltes, 1994; Lin et al., 2013; Heywood et al., 2017; Huh, 2018; Jayakody et al., 2018; Loughrey et al., 2018). Our current results are consistent with previous investigations. Compared with normally hearing subjects, patients with long-term bilateral hearing loss exhibited significantly poor cognitive performances in MMSE, VF, BDS, TMTA, SCWTA, SCWTB, and SCWTC tests.

Hearing loss contributes to changes in the brain network associated with cognitive processing. Resting-state fMRI studies reported increased functional connectivity and abnormal neural activities in the default mode network (DMN) of individuals with hearing loss compared with normally hearing subjects (Husain et al., 2014; Xu et al., 2017). In addition, previous studies also reported decreased resting-state functional connectivity from the insula to the DMN (Xu et al., 2019a,d), as well as reduced thalamic connectivity with the DMN (Xu et al., 2019c). These investigations indicate that hearing loss leads to neural activity changes in the DMN, which is responsible for cognitive processing (Wheeler et al., 2006; Leech et al., 2011). However, it is not entirely clear whether changes in brain networks contribute to cognitive decline in hearing loss patients. This current study found that the right dorsolateral prefrontal cortex (BA 9R), left subgenual cortex (BA 25L), left cingulate cortex (BA 30L), and the associative visual cortex presented significantly weakened asynchronous causal connections with those brain regions associated with cognitive, language, auditory, and sensor motor processing. Moreover, BAs 9R, 25L, 30L, and 19L presented significantly reduced nodal degrees, which were associated with poor cognitive performances. Previous studies indicate that BA 9R plays a crucial role in visual processing, working memory, and the decisional processes (Manoach et al., 1999; Pierrot-Deseilligny et al., 2003); BA 25L is involved in cognitive behavior activity associated with emotional process (Drevets et al., 1997); BA 30L is associated with implementing retrieval strategies for episodic memory (Nestor et al., 2003); and the associative visual cortex is associated with visual processing, associative memory formation, and cognitive function (McKee et al., 2006; Gazzaley et al., 2007; Rosen et al., 2018). These results suggest that changes in the causal connectivity network might be an important reason that contributes to cognitive decline in hearing loss patients.

In a word, our findings suggest that changes in brain causal connectivity networks are an important mark of cognitive decline in patients with hearing loss. Auditory and visual language signals might have different effects on speech processing. Our research provides some implications for rehabilitation of patients with hearing loss.

### Limitations

Our study has a lower limit of sample size; therefore, significant effects observed may also be the consequence of small power. Hearing loss during development and hearing loss in adulthood may have different effects. The aim of the present study was to investigate adult-onset long-term bilateral mild-to-severe sensorineural hearing loss. However, the use of auditorily presented cognitive tests might have biased the results. In addition, we only investigated resting-state causal connectivity, and these results might be different with stimulus-related connectivity (Okano et al., 2020; Yusuf et al., 2021).

## Data Availability Statement

The original contributions presented in the study are included in the article/supplementary material, further inquiries can be directed to the corresponding author.

## Ethics Statement

The studies involving human participants were reviewed and approved by The Institutional Ethics Committee of Shandong First Medical University. The patients/participants provided their written informed consent to participate in this study.

## Author Contributions

G-YZ and L-CX designed the general approach and wrote the manuscript. W-BZ, GZ, and YZ collected the clinical data. M-FZ and L-CX collected the MRI data. G-YZ, Y-FC, D-SZ, and L-MH processed the MRI data. P-CW and X-YW advised on the experimental design. GZ, P-CW, and X-YW revised the manuscript. All authors contributed to the article and approved the submitted version.

## Conflict of Interest

The authors declare that the research was conducted in the absence of any commercial or financial relationships that could be construed as a potential conflict of interest.

## Footnotes

1 http://www.fil.ion.ucl.ac.uk/spm/software/spm8/

2 https://www.nitrc.org/projects/vdb/
